# Rotavirus P[4]G2 in a Vaccinated Population, Brazil

**DOI:** 10.3201/eid1405.071426

**Published:** 2008-05

**Authors:** Alexandre C. Linhares, F. Raúl Velázquez

**Affiliations:** * Ministério da Saúde, Belém, Pará, Brazil; † Instituto Mexicano del Seguro Social, Mexico City, Mexico

**Keywords:** rotavirus, vaccine, G2, letter

## To the Editor

 Gurgel et al. described the predominance of P[4]G2 rotaviruses in a vaccinated population in Aracaju, northeastern Brazil (*1*). However, several limitations need to be addressed to avoid misinterpretation of data that could lead to loss of confidence in the vaccine in Brazil and other countries.

Brazil was one of the first countries in Latin America to introduce a live, oral, attenuated human rotavirus vaccine into a public-sector health program. Nevertheless, vaccine coverage levels vary considerably across regions (≈40% to >80%) and are ≈50% in some parts of northern and northeastern Brazil. Therefore, drawing conclusions about the vaccine’s protection and prevailing rotavirus genotypes in a setting where coverage is still low seems premature.

Two findings require special consideration. First, although the number of patients is small, children <1 year of age showed a reduced risk for severe rotavirus diarrhea among vaccinated (7%) patients compared with nonvaccinated (26%) patients: p<0.05; odds ratio (OR) 0.20; exact 95% confidence interval (CI) 0.029–1.24. Second, surveillance was conducted for only 4 months, which did not allow for demonstration of a true representative pattern of strain distribution over time. The sequential changing predominance of rotavirus serotypes occurring over time has been well documented for many years (*2*).

The authors stated that the “vaccine does not afford complete protection against infection” (*1*). For those not paying close attention to data analysis, this statement could be misinterpreted to mean that the vaccine may not protect against P[4]G2. To the contrary, even with a small sample size and low vaccine coverage, additional analyses of the original data show that the live, oral, attenuated human rotavirus vaccine can protect against the 100% predominance of P[4]G2.

In a large phase III trial conducted in Latin America and Finland, a nonsignificant but protective trend was observed against severe disease associated with P[4]G2 (*3*). Furthermore, in a subsequent meta-analysis, protection against P[4]G2 rotavirus gastroenteritis of any severity was 81% (95% CI 31–96) and protection against severe rotavirus gastroenteritis was 71% (95% CI 20–91) (*4*).

To reinforce the hypothesis that predominance of P[4]G2 strains in Aracaju is unrelated to vaccine use, it is worth mentioning that P[4]G2 rotaviruses appear to display an ≈10-year cyclic pattern of occurrence in Brazil (*5*). Although the data presented in the original article may cause misinterpretation about vaccine protection, the article highlights the need for well-designed postmarketing studies to assess both vaccine impact and strain surveillance, in compliance with recent World Health Organization recommendations (*6*).
