# Supplementary material for: Rotavirus P[4]G2 in a Vaccinated Population, Brazil
Source: Emerg Infect Dis. 2008 May;14(5):864. doi: 10.3201/eid1405.071426 (PMC2600233; doi:10.3201/eid1405.071426)
Supplement: Related texts

# Reply: In Response

**Authors:** Ricardo Queiroz Gurgel, Sarah Cristina Fontes Vieira, Vanessa Cristiane Farias Barros, Paula Brandão Fontes, Eduardo F. Salustino, Osamu Nakagomi, Toyoko Nakagomi, Winifred Dove, Nigel A. Cunliffe, Luis E. Cuevas

**Affiliations:** * Federal University of Sergipe, Aracaju, Brazil; † University of Liverpool, Liverpool, UK; ‡ Nagasaki University, Nagasaki, Japan; § Liverpool School of Tropical Medicine, Liverpool, UK

 We acknowledge the comments by Patel et al. (*1*) and by Linhares and Velázquez (*2*) about our article that documented the presence of a single rotavirus genotype (P[4]G2) in Aracaju, northeastern Brazil, after the introduction of a human, monovalent rotavirus vaccine (*3*). Both letters emphasize that the predominance of P[4]G2 may be caused by a natural genotype variation unrelated to vaccination. We agree that our observation could be explained by natural variation of circulating rotavirus genotypes in the region, but an alternative possibility is that the introduction of the G1P[8] rotavirus vaccine into the childhood immunization schedule created conditions in which P[4]G2 strains had a selective advantage over strains with which the vaccine shares G type, P type, or both.

According to a systematic review of rotavirus genotypes reported in the 25 years preceding introduction of the vaccine in Brazil, the prevalence of P[4]G2 strains varied from 19% (1986–1995) to 12% (1996–2000) to 1% thereafter, thus not reaching the detection rate we observed in Aracaju (R.Q. Gurgel et al., unpub data). Furthermore, in the ensuing 8-month period, no genotype other than P[4]G2 had been detected in Aracaju, suggesting that our initial findings were not spurious (R.Q. Gurgel et al., unpub data). In addition, in a separate study we conducted in Recife, a city 500 km north of Aracaju, we observed a significant increase in the proportion of G2 strains detected from 47% (21/45) during the 3-month period immediately after vaccine introduction (March 2006–May 2006) to 100% (11/11) during the same 3-month period 1 year after the vaccine introduction (March 2007–May 2007) (*4*). We believe that our findings are consistent with results of field trials that indicated that the vaccine provided relatively less protection against P[4]G2 strains than against other rotavirus strain types (*5*).

The beneficial impact of rotavirus vaccination in northeastern Brazil is reflected in the reduction of the detection rate of rotavirus among severe diarrhea cases in our study in Recife, which fell from 27% (45/166 cases) to 5.0% (11/221 cases) in the postvaccine 3-month reporting periods, respectively (*4*). Our data from Aracaju are indicative of heterotypic protection, although this is not statistically significant (*1*), against P[4]G2 strains. Further postlicensure studies in Brazil are required to document continuing effectiveness of the national vaccination program as well as to closely monitor the circulating rotavirus strain types (*6*).

## References

[1] Patel MM, de Oliveira LH, Bispo AM, Gentsch J, Parashar UD. Rotavirus P[4]G2 in a vaccinated population, Brazil [letter]. Emerg Infect Dis. 2008;14:863.1843939010.3201/eid1405.071440PMC2600230

[2] Linhares AC, Velázquez FR. Rotavirus P[4]G2 in a vaccinated population, Brazil [letter]. Emerg Infect Dis. 2008;14:864.10.3201/eid1405.071440PMC260023018439390

[3] Gurgel RQ, Cuevas LE, Vieira SCF, Barros VCF, Fontes PB, Salustino ET, Predominance of rotavirus P[4]G2 in a vaccinated population, Brazil. Emerg Infect Dis. 2007;13:1571–3.1825801110.3201/eid1310.070412PMC2851506

[4] Nakagomi T, Cuevas LE, Gurgel RQ, Elrokhsi SH, Belkhir YA, Abugalia M, Apparent extinction of non-G2 rotavirus strains from circulation in Recife, Brazil, after the introduction of rotavirus vaccine. [PMID: 18175037]. Arch Virol. 2008;153:591–3. 10.1007/s00705-007-0028-z18175037

[5] Ruiz-Palacios GM, Perez-Schael I, Velazquez FR, Abate H, Breuer T, Clemens SC, Safety and efficacy of an attenuated vaccine against severe rotavirus gastroenteritis. N Engl J Med. 2006;354:11–22. 10.1056/NEJMoa05243416394298

[6] Rotavirus vaccines. Wkly Epidemiol Rec. 2007;82:285–96.17691162
